# Gender-specific association of blood lipids and reproductive trajectory with cognitive impairment: A community based cross-sectional study from India

**DOI:** 10.3389/fpsyg.2023.1107152

**Published:** 2023-02-27

**Authors:** Kevingu Khate, Vineet Chaudhary, Imnameren Longkumer, Kallur Nava Saraswathy, Naorem Kiranmala Devi

**Affiliations:** Department of Anthropology, University of Delhi, New Delhi, India

**Keywords:** sex-specific, cognitive impairment, lipids, HDL-cholesterol, menopause

## Abstract

**Background:**

Abnormal blood lipid levels in the general population and adverse reproductive events among women have been associated with cognitive impairment (CI). However, their relationship has not been extensively studied in community settings. Hence, this study aims to explore the association of CI with blood lipid levels in both sexes and reproductive events/trajectory among women.

**Methods:**

A cross-sectional study was conducted among a North Indian rural population. A total of 808 adults were recruited through door-to-door household survey. Data on socio-demographic variables, reproductive profile of women, and cognitive impairment status were collected. Fasting blood sample was collected to estimate serum lipid profile. Multivariate logistic regression was performed to test for association.

**Results:**

The study demonstrated a lack of association between lipid profile and cognitive impairment among males. Surprisingly, low HDL-C among females was found to be protective against moderate/severe cognitive impairment (value of *p* = 0.049). Further, menopausal women and those having five or higher live births were found to be at higher risk of CI than pre-menopausal women and those with 1–2 live births, respectively.

**Conclusion:**

The present study hints toward a gender-specific association of blood lipid levels with CI. Further, higher live births and menopause appear to be important risk factors for CI among women.

## Introduction

Cognitive impairment (CI) can range from mild cognitive impairment (MCI), which is commonly regarded as the transition stage between an expected cognitive decline due to aging and dementia, to more advanced and serious forms of dementia, such as Alzheimer’s disease (Pankratz et al., 2015). The global prevalence of cognitive impairment was estimated to range between 1 and 42% depending on the setting (clinical vs. population-based) and classification used (Ward et al., 2012; Sachdev et al., 2015; Petersen, 2016). In India, various studies have reported wide variation in the prevalence of MCI among different populations ranging from 3.5 to 63.2% (Das et al., 2007; Kumar and Sudhakar, 2013; Sharma et al., 2013; Sengupta et al., 2014; Khullar et al., 2017; Kaur et al., 2018).

Since MCI in itself is a transient phase, not all individuals experiencing MCI progress to dementia (Aretouli et al., 2010; Ganguli et al., 2011). While some MCI patients might develop advanced dementia, others may regain normal cognitive function with time (Aretouli et al., 2010; Ganguli et al., 2011) and intervention (Meng et al., 2021). Though studies have acknowledged the complexity of defining loss of cognitive functions, especially the subclinical expressions of CI, into discrete categories (Ritchie et al., 2001; Richardson et al., 2019), cognitive impairment, for research purposes, has often been classified into three categories, i.e., mild, moderate and severe CI (Murray et al., 2006; Choe et al., 2008).

Cognitive impairment is etiologically complex. Apart from socio-demographic parameters like gender, education level, occupational status, and family medical history, several neurologic, systemic, genetic, biochemical, and psychiatric factors are believed to contribute to the development and progression of CI (Lopez, 2013; Singh et al., 2023). Of these, abnormal lipid levels and hypercholesterolemia are emerging as important modifiable risk factors for CI and other psychiatric conditions (Cicconetti et al., 2004; Khullar et al., 2017; Chaudhary et al., 2022). Further, as estrogen levels are known to play a vital role in neurocognitive function, adverse reproductive history/events and hormonal imbalances are also being investigated for their role in cognitive decline among women (Li et al., 2016; Shimizu et al., 2019; Song et al., 2020).

Despite clinical reports linking blood lipid levels and reproductive trajectory with cognitive health, the relationship between cognitive impairment and lipid levels or women’s reproductive trajectory in community settings remains understudied. Since blood lipid levels, as well as reproductive trajectories, are greatly affected by the ecological and cultural milieu of a community, community-specific studies exploring the relationship between CI and blood lipids or CI and women’s reproductive trajectory are crucial to designing effective interventions. Looking at the paucity of community-based studies, especially from India, the present study aims to explore the association of CI with blood lipid levels in both sexes and reproductive events/trajectory among women of an endogamous North Indian Population.

## Materials and methods

### Participant selection and data collection

A cross-sectional study design was adopted for the present study. A total of 808 individuals of both sexes (334 male and 474 females), aged 30–70 years (median age 52 years), belonging to Jat community of Palwal district, Haryana, North India, were conveniently recruited. The participants constituted a Mendelian population, sharing a common gene pool where marriages are mostly within the same community. Individuals fulfilling above criteria, with self-reported absence of physical and mental illnesses, were recruited through door-to-door household survey. Individuals suffering from chronic diseases (e.g., cancers and CVDs), those on long-term medications, and pregnant and lactating mothers were excluded from the study.

Data pertaining to socio-demographic variables (age, literacy, occupation, family income, and marital status) were collected using pretested interview schedules. Reproductive profile/trajectory (menopausal status, number of conceptions, number of live births, miscarriage, age at menarche, age at menopause, and years since menopause) among females were also obtained using a standardized questionnaire.

The study was approved by the Institutional Ethics Committee, Department of Anthropology, University of Delhi (Ref No. Anth/2018/2890/1/28-12-2018). Pre-informed written voluntary consent, transcribed in local language, was obtained from each participant prior to recruitment and data collection.

### Cognitive assessment

The cognitive status of the participants was assessed using Mini-Mental State Examination (MMSE) scale (Folstein et al., 1975). MMSE is a cross-culturally validated and widely used 30-point tool for the assessment of cognitive functions (Folstein et al., 1975). Each item of the MMSE was administered to the participant as per the instruction manual by trained field workers, and the responses were carefully noted. Based on the responses, each participant was scored out of 30. Individuals with scores ≥24 were considered to have normal cognition; those with scores between 19 and 23 were considered to have mild cognitive impairment (CI), while those with 10–18 and 0–9 were considered to have moderate and severe CI, respectively. Due to the extremely low prevalence of severe CI (*n* = 2), the severe CI category was merged with the moderate CI category for various statistical analyses.

### Lipid measurements

Lipid profile, which included total cholesterol (TC), triglyceride (TG), and high-density lipoprotein cholesterol (HDL-C), was estimated from serum extracted from overnight fasting blood (~12 h of fasting) using commercial kits (Randox Laboratories Ltd.). Low-density lipoprotein cholesterol (LDL-C) and very low-density lipoprotein cholesterol (VLDL-C) were calculated using Friedewald and Fredrikson’s formula (Friedewald et al., 1972). Normal levels of blood lipids were defined as TC < 200, TG < 150, HDL-C ≥ 40 (for males), and ≥50 mg/dL (for females), LDL-C < 130, and VLDL-C < 30 mg/dL (Expert Panel on Detection, Evaluation, and Treatment of High Blood Cholesterol in Adults, 2001).

### Statistical analysis

Statistical analyses were performed using IBM SPSS ver.22 software. The Kolmogorov–Smirnov test was used to determine if a continuous variable is normally distributed. Medians along with respective interquartile ranges (IQR) have been reported for non-normally distributed continuous variables, and numbers with respective percentages have been reported for categorical variables. Mann–Whitney *U*-test, Kruskal–Wallis, and chi-square test (as appropriate) were used to determine significant differences between various groups. Furthermore, logistic regression analysis was performed to understand the association between the dependent (cognitive impairment) and independent variables (lipid levels and reproductive trajectory). Confounders such as age, education, and employment status were identified and controlled while calculating the odds ratio. All statistical tests were considered significant at value of *p* < 0.05.

## Results

### General characteristics of participants

The distribution of sociodemographic variables (age, education status, occupation status, family income, and marital status) among individuals with normal cognition and those with mild or moderate/severe CI revealed that the proportions of individuals without any formal education or unemployed individuals among both males and females and older individuals among females were significantly higher in the mild and moderate/severe CI categories when compared to normal cognition category (Table 1). These variables were considered confounders while calculating the odds ratio.

**Table 1:** Sex-wise distribution of sociodemographic variables with respect to cognitive status.

		Males (*N* = 334)				Females (*N* = 474)			
		Normal (*N* = 217) *n* (%)	Mild CI (*N* = 84) *n* (%)	Mod/Sev CI (*N* = 33) *n* (%)	Value of *p*	Normal (*N* = 81) *n* (%)	Mild CI (*N* = 192) *n* (%)	Mod/Sev CI (*N* = 201) *n* (%)	Value of *p*
Median age (IQR) (in years)		54 (46–62)	57.50 (48.25–63.00)	59 (50.50–69.00)	0.079	42 (38–48)	50 (43–58)	54 (45.50–61.50)	<0.001^*^
Education status	Having formal education	207 (95.4)	59 (70.2)	5 (15.2)	<0.001^*^	62 (76.5)	40 (20.8)	16 (8.0)	<0.001^*^
	No formal education	10 (4.6)	25 (29.8)	28 (84.8)		19 (23.5)	152 (79.2)	185 (92.0)	
Employment status	Employed	73 (33.6)	12 (14.3)	1 (3.0)	<0.001^*^	4 (4.9)	2 (1.0)	1 (0.5)	0.016^*^
	Unemployed	144 (66.4)	72 (85.7)	32 (97.0)		77 (95.1)	190 (99.0)	200 (99.5)	
Annual family income (INR)	<50,000	28 (12.9)	8 (9.5)	3 (9.1)	0.635	11 (13.6)	12 (6.3)	22 (10.9)	0.110
	≤50,000	189 (87.1)	76 (90.5)	30 (90.9)		70 (86.4)	180 (93.8)	179 (89.1)	
Marital status	Married	203 (93.5)	77 (91.7)	29 (87.9)	0.097	74 (91.4)	176 (91.7)	185 (92.0)	0.934
	Unmarried	4 (1.8)	2 (2.4)	4 (12.1)		0 (0.0)	2 (1.0)	0 (0.0)	
	Widowed	10 (4.6)	5 (6.0)	0 (0.0)		7 (8.6)	14 (7.3)	16 (8.0)	

* Significant at value of *p* < 0.05;

*N* = sample size of the category/sub group; *n* = count; Normal, normal cognition; CI, cognitive impairment; Mod/Sev, moderate or severe; IQR, inter quartile range; INR, Indian rupees.

### Median blood lipid levels and cognition status

The overall distribution of median blood lipid levels with respect to cognitive status revealed that median TG and VLDL-C levels were significantly higher among individuals in the normal category compared to those individuals with mild CI (Table 2). However, after stratification for sex, the trend of higher TG in the normal cognition category than in the mild CI category remained significant only among females (Table 2). On the other hand, in the overall analysis, the median HDL-C level was found to be significantly higher among individuals in the moderate/severe CI group compared to those in the normal cognition group (Table 2). This trend lost statistical significance in the sex-stratified analysis.

**Table 2:** Overall and sex-stratified distribution of median blood lipid levels with respect to cognitive status.

	Overall (*N* = 808)			Males (*N* = 334)			Females (*N* = 474)			
	Normal median (IQR) (*N* = 298)	Mild CI median (IQR) (*N* = 276)	Mod/Sev CI median (IQR) (*N* = 234)	Normal median (IQR) (*N* = 217)	Mild CI median (IQR) (*N* = 84)	Mod/Sev CI median (IQR) (*N* = 33)	Normal median (IQR) (*N* = 81)	Mild CI median (IQR) (*N* = 192)	Mod/Sev CI median (IQR) (*N* = 201)	Value of *p*
TC	175.52 (145.75–209.78)	173.26 (148.52–204.80)	175.22 (150.00–204.48)	176.75 (145.00–211.71)	173.88 (145.51–206.19)	180.54 (160.57–213.70)	172.30 (150.69–202.28)	172.43 (150.10–202.00)	174.32 (149.23–203.74)	0.776^α^ 0.581^β^ 0.978^γ^
TG	111.21 (75.16–164.24)	96.08 (72.28–134.05)	109.95 (74.81–146.21)	120.90 (83.22–166.25)	112.78 (84.57–145.44)	92.12 (68.41–147.66)	84.16 (66.20–134.75)	89.91 (67.96–125.10)	111.87 (76.35–146.44)	0.017^α,*^ 0.099^β^ 0.008^γ,*^
HDL-C	46.72 (38.00–55.68)	48.32 (40.64–55.42)	51.20 (40.96–61.72)	46.40 (36.80–56.08)	43.68 (36.16–53.30)	52.37 (40.16–65.41)	47.36 (41.60–53.80)	49.76 (42.40–56.40)	50.66 (40.90–61.61)	0.001^α,*^ 0.060^β^ 0.196^γ^
LDL-C	106.67 (77.04–133.81)	102.35 (81.13–127.48)	99.71 (76.63–128.70)	108.42 (71.94–135.70)	104.61 (80.80–127.66)	110.62 (80.89–132.91)	105.91 (83.14–128.60)	100.61 (81.18–128.61)	98.08 (75.57–126.67)	0.784^α^ 0.901^β^ 0.597^γ^
VLDL-C	21.91 (14.99–32.73)	19.0 (14.45–26.69)	21.89 (14.91–29.12)	23.80 (16.61–33.20)	22.55 (16.91–29.08)	18.18 (12.65–28.92)	16.83 (13.24–26.95)	17.96 (13.59–25.01)	22.37 (15.27–29.28)	0.019^α,*^ 0.046^β,*^ 0.006^γ,*^

^*^Significant at value of *p* < 0.05; α = overall; β = males; γ = females; TC, total cholesterol; TG, triglycerides; HDL-C, high density lipoprotein cholesterol; LDL-C, low density lipoprotein cholesterol; VLDL-C, very low density lipoprotein cholesterol; Normal, normal cognition; CI, cognitive impairment; Mod/Sev, moderate or severe; IQR, inter quartile range; *N* = sample size of the category/sub group.

### Odds ratio analyses exploring the association between CI and abnormal blood lipid levels

Sex-stratified adjusted binary logistic regression models suggested a gender-specific relationship between blood lipid parameters and cognition status (Table 3). While none of the studied lipid variables were found to be associated with CI among males, females with low HDL-C were found to be at a reduced risk for moderate/severe cognitive impairment when compared to those with normal HDL-C levels (OR = 0.517; 95% CI = 0.268–0.996; value of *p* = 0.049; Table 3); however, since the observed value of *p* (=0.049) was close to the threshold value of *p* of 0.05, the finding was considered to have suggestive significance, and further analysis was taken up (with respect to HDL-C quartiles) to validate (or rule out) this finding.

**Table 3:** Sex-stratified binary logistic regression analyzes exploring the association between cognitive impairment and abnormal blood lipid levels (with normal blood levels as the reference).

Variables	Males (*N* = 334)			
	Mild CI (*N* = 84)		Mod/Sev CI (*N* = 33)	
	OR^α^ (95% confidence interval)	Value of *p*	OR^α^ (95% confidence interval)	Value of *p*
High TC	0.871 (0.477–1.589)	0.652	0.892 (0.269–2.962)	0.852
High TG	0.578 (0.306–1.091)	0.091	0.748 (0.204–2.749)	0.662
Low HDL-C	0.773 (0.434–1.374)	0.380	0.487 (0.142–1.673)	0.253
High LDL-C	0.679 (0.355–1.297)	0.241	0.699 (0.192–2.326)	0.527
High VLDL-C	0.586 (0.310–1.106)	0.099	0.657 (0.171–2.519)	0.540
	Females (*N* = 474)				Mild CI (*N* = 192)		Mod/Sev CI (*N* = 201)	
OR^α^ (95% confidence interval)	Value of *p*	OR^α^ (95% confidence interval)	Value of *p*
High TC	0.739 (0.356–1.535)	0.417	0.701 (0.301–1.636)	0.412
High TG	0.788 (0.351–1.767)	0.562	0.594 (0.234–1.511)	0.274
Low HDL-C	0.604 (0.319–1.144)	0.122	0.517 (0.268–0.996)	0.049^*^
High LDL-C	0.951 (0.451–2.003)	0.895	0.673 (0.275–1.646)	0.386
High VLDL-C	0.764 (0.338–1.724)	0.517	0.594 (0.234–1.511)	0.274

* Significant at value of *p* < 0.05; α = adjusted for age (among females only), education status and employment status; OR, odds ratio; Normal, normal cognition; CI, cognitive impairment; Mod/Sev, moderate or severe; *N* = sample size of the category/sub group.

### Prevalence of CI in quartiles of HDL-C

Prompted by this rather surprising finding (reduced risk of CI in low HDL-C category among females), the study sample was divided into four categories with respect to HDL-C quartiles to determine the prevalence of CI in various quartiles. This analysis revealed that, among females, the prevalence of moderate/severe CI was highest in the fourth HDL-C quartile, followed by the first, second, and third quartiles (Table 4). This difference in the distribution of individuals with moderate/severe CI in various HDL-C quartiles among females was statistically significant. No such statistically significant trend was observed among males (Table 4).

**Table 4:** Prevalence of cognitive impairment in various HDL-C quartiles.

	HDL-C quartiles (in mg/dL)	Normal *n* (%)	Mild CI *n* (%)	Mod/Sev CI *n* (%)	Value of *p*
Males, *N* = 334	1st (<37.1)	55 (26.1)	23 (27.4)	5 (15.2)	0.250
	2nd (37.1–45.3)	49 (23.2)	26 (31.0)	6 (18.2)	
	3rd (45.3–56.1)	54 (25.6)	19 (22.6)	9 (27.3)	
	4th (>56.1)	53 (25.1)	16 (19.0)	13 (39.4)	
Females, *N* = 474	1st (<41.6)	20 (25.3)	44 (23.7)	54 (27.3)	0.002^*^
	2nd (41.6–49.6)	26 (32.9)	49 (26.3)	40 (20.2)	
	3rd (49.6–57.8)	23 (29.1)	54 (29.0)	38 (19.2)	
	4th (>57.8)	10 (12.7)	39 (21.0)	66 (33.3)	

* Significant at value of *p* < 0.05; *N* = sample size of the category; *n* = count; Normal, normal cognition; CI, cognitive impairment; Mod/Sev, moderate or severe.

Looking at the significant difference in the distribution of moderate/severe CI in various quartiles of HDL-C among females (lower frequency of moderate/severe CI in middle quartiles and higher in the extreme quartiles), odds ratio analysis (with second and third quartiles as the reference) was performed to understand the risk of CI in the first, fourth and combined first and fourth quartiles (Table 5). This analysis revealed that those in the fourth quartile or in the combined first + fourth quartile were at significantly higher risk of moderate/severe CI than those in the middle quartile (Table 5).

**Table 5:** Logistic regression showing the risk of cognitive impairment in the lowest and the highest quartiles of HDL-C (with middle quartiles as the reference) among females.

HDL-C quartiles	Mild CI (*N* = 192)		Mod/Sev CI (*N* = 201)	
	OR (95% confidence interval)	Value of *p*	OR^α^ (95% confidence interval)	Value of *p*
1st vs. 2nd + 3rd	0.778 (0.373–1.622)	0.502	1.193 (0.549–2.592)	0.656
4th vs. 2nd + 3rd	2.146 (0.892–5.167)	0.088	4.945 (1.984–12.325)	0.001^*^
1st + 4th vs. 2nd + 3rd	1.168 (0.629–2.170)	0.623	2.194 (1.140–4.224)	0.019^*^

* Significant at value of *p* < 0.05; CI, cognitive impairment; Mod/Sev, moderate or severe; OR, odds ratio; *N* = sample size of the category.

### Reproductive trajectory and cognitive impairment

The distribution of reproductive variables was seen among the groups of women with different cognitive statuses (normal cognition vs. mild CI vs. moderate/severe CI; Table 6). The reproductive events included were menopausal status, number of conceptions, number of live births, miscarriage, age at menarche, age at menopause, and years since menopause. No significant differences in the distribution of the above-mentioned reproductive events were found among the groups of women with different cognitive statuses, except for menopausal status and the number of live births, where the proportions of menopausal women and those having five or more live births were significantly higher in the moderate/severe CI category than the normal cognition category.

**Table 6:** Relationship between reproductive events and cognitive impairment among women participants.

		Normal *n* (%)	Mild CI *n* (%)	Mod/Sev CI *n* (%)	Value of *p*	Mild CI OR (95% confidence interval)	Mod/Sev CI OR (95% confidence interval)
Menopausal status (*N* = 340)	Pre-menopause	34 (55.7)	44 (30.6)	23 (17.0)	<0.001^*^	Reference	Reference
	Natural menopause	17 (27.9)	78 (54.2)	93 (68.9)		3.55 (1.78–7.07)^*^	8.09 (3.86–16.95)^*^
	Hysterectomy	10 (16.4)	22 (15.3)	19 (14.1)		1.70 (0.71–4.06)	2.80 (1.10–7.12)^*^
Number of conceptions (*N* = 248)	0	2 (4.3)	6 (5.8)	2 (2.0)	0.201	1.87 (0.27–13.20)	0.83 (0.08–8.24)
	1–2	5 (10.6)	8 (7.8)	6 (6.1)		Reference	Reference
	3–4	28 (59.6)	54 (52.4)	44 (44.9)		1.21 (0.36–4.03)	1.31 (0.36–4.70)
	≥5	12 (25.5)	35 (34.0)	46 (46.9)		1.82 (0.50–6.66)	3.19 (0.83–12.28)
Number of live births (*N* = 229)	0	1 (2.3)	0 (0.0)	0 (0.0)	0.016^*^	0.33 (0.01–9.26)	0.49 (0.02–13.92)
	1–2	9 (20.9)	9 (9.4)	6 (6.7)		Reference	Reference
	3–4	27 (62.8)	64 (66.7)	53 (58.9)		2.37 (0.84–6.62)	2.94 (0.95–9.14)
	≥5	6 (14.0)	23 (24.0)	31 (34.4)		3.83 (1.06–13.91)^*^	7.75 (2.00–29.99)^*^
Miscarriage (*N* = 208)	No	33 (89.2)	66 (79.5)	77 (87.5)	0.245	Reference	Reference
	Yes	4 (10.8)	17 (20.5)	11 (12.5)		2.13 (0.66–6.82)	1.18 (0.35–3.97)
Age at menarche (in years; *N* = 431)	≤12	48 (65.8)	127 (70.6)	108 (60.7)	0.388	1.32 (0.31–5.50)	0.68 (0.18–2.56)
	13–15	3 (4.1)	6 (3.3)	10 (5.6)		Reference	Reference
	≥16	22 (30.1)	47 (26.1)	60 (33.7)		1.07 (0.24–4.67)	0.82 (0.21–3.25)
Age at menopause (in years; *N* = 286)	<40	19 (57.6)	62 (53.9)	71 (52.2)	0.678	1.48 (0.45–4.81)	0.93 (0.31–2.82)
	40–50	5 (15.2)	11 (9.6)	20 (14.7)		Reference	Reference
	>50	9 (27.3)	42 (36.5)	45 (33.1)		2.12 (0.59–7.62)	1.25 (0.37–4.21)
Years since menopause (in years; *N* = 239)	0–3	2 (7.4)	9 (9.0)	7 (6.3)	0.679	Reference	Reference
	4–6	5 (18.5)	18 (18.0)	14 (12.5)		0.80 (0.13–4.96)	0.80 (0.12–5.21)
	7–10	8 (29.6)	20 (20.0)	23 (20.5)		0.56 (0.10–3.16)	0.82 (0.14–4.80)
	>10	12 (44.4)	53 (53.0)	68 (60.7)		0.98 (0.19–5.14)	1.62 (0.30–8.75)

* Significant at value of *p* < 0.05; *N* = sample size of the category; *n* = count; Normal, normal cognition; CI, cognitive impairment; Mod/Sev, moderate or severe; OR, odds ratio.

In odds ratio analysis, menopausal women (with pre-menopausal category as reference) were found to be at 3.5 and 8-fold significantly higher risk of mild CI and moderate/severe CI, respectively, (value of *p* < 0.05). Further, hysterectomized women were also found to be at a 2.8-fold significantly increased risk of moderate/severe CI than pre-menopausal women. Again, women with five or more live births (with 1–2 live birth as the reference category) were at 3.8 and 7.7-fold significantly increased risk of mild CI and moderate/severe CI, respectively, (value of *p* < 0.05; Table 6). Other reproductive events were not found to be associated with CI status.

## Discussion

Abnormal lipid levels and adverse reproductive events have been reported to influence cognitive functions and play a role in the development of cognitive impairments (Van Exel et al., 2002; Devore et al., 2004; Power et al., 2018; Lv et al., 2019; Ning et al., 2020; Song et al., 2020; Gong et al., 2022). However, very few studies have methodically investigated their relationship in community settings. Further, even lesser attention has been given to the gender-specific relationship between blood lipids and CI (Ancelin et al., 2013, 2014; Svensson et al., 2019; Choe et al., 2021; Bakeberg et al., 2021a). It is important to highlight that sex is a crucial factor to be considered when conducting cognitive assessment studies (Reekes et al., 2020; Bakeberg et al., 2021a). Literature suggests that women have better verbal and non-verbal reasoning skills, such as language, fluency, memory, and decision-making, when compared to their male counterparts (Li and Singh, 2014). Other studies have also emphasized on sex-specific approach to studying CI because of physiological differences between males and females, which eventually cause cerebrovascular and cognitive decline (Miller et al., 2013; Reekes et al., 2020; Sundermann et al., 2021). Considering these research gaps, the present study attempted to investigate the relationship between blood lipid levels and CI among both males and females and reproductive trajectory and CI among females of an endogamous North Indian Population.

In the present study, median TG and VLDL-C levels were significantly higher among individuals with normal cognition than those with mild CI. This observation is in concordance with some of the previous reports where TG levels have been reported to be significantly higher in control groups (normal cognition group) than in case groups (MCI group; Dimopoulos et al., 2007; Lepara et al., 2009; He et al., 2016). However, contrary findings have also been reported (De Frias et al., 2007; Sims et al., 2008). Since the adverse implications of pathologically high TG can easily outweigh the benefits of high TG in cognitive functions, population-based studies, as well as clinical trials, are warranted to establish population-specific healthy ranges of TG. Again, despite significant differences in the median TG levels between normal cognition and mild CI groups, adjusted logistic regression models failed to find any significant association between TG and CI (in overall as well as sex-stratified analyses). Further research is required to explicate the relationship between TG and CI.

In the present study, the median HDL-C level was found to be higher among individuals with moderate/severe CI than those with normal cognition. In odds ratio analysis, low HDL-C was found to be protective against CI among females but not among males. Also, females in the fourth quartile of HDL-C were at significantly increased risk of CI than those in the second and third quartiles. Overall, these observations hint toward low HDL-C being protective and high HDL-C being a risk for CI among females (suggestive significance). Though these findings are largely in contradiction with previous reports (Reitz et al., 2010; Hottman et al., 2014; Vitali et al., 2014; Bates et al., 2017), Ancelin et al. (2014), in their study among elderly individuals, reported an increased risk of decline in cognitive functions among females having high HDL-C than those having low HDL-C. A recent study conducted among females with Parkinson’s disease (PD) has also found elevated HDL-C to significantly contribute to cognitive decline (Bakeberg et al., 2021b). The mechanism behind the higher risk of CI among women having higher HDL-C remains to be clarified, yet, studies have suggested the role of genetic vulnerability related to HDL-C, estrogen receptors, or metabolizing enzymes that could affect HDL-C responsiveness (Ancelin et al., 2014).

Other lipid variables, *viz.* TC and LDL-C, were not found to be associated with CI in either sex. Previous studies investigating the role of plasma lipid levels in cognitive functions have reported inconsistent findings (Mielke et al., 2005; Reitz et al., 2008; Morley and Banks, 2010; Li et al., 2018). The relationship between plasma lipids and cognitive function, the role of gender in modulating this relationship, as well as the mechanism by which plasma lipids affect cognitive function are still not fully understood.

Coming to the role of reproductive trajectory in cognitive impairment, menopausal women and women with five or more live births were found to be at a higher risk of CI. Previous studies have also reported menopause (Weber et al., 2014; Conde et al., 2021) and higher numbers of children (Beeri et al., 2009; Read and Grundy, 2017) as risk factors for cognitive decline.

Women have been shown to experience cognitive deficiencies during the menopausal transition, notably in areas like working memory, attention, slower processing speeds, and verbal memory (Miller and Cronin-Golomb, 2010; Lin et al., 2018). There is growing evidence that oestrogen has a major neuroprotective and neurotrophic effect on the central nervous system (Conde et al., 2021). The decline in oestrogen levels among post-menopausal women can be one of the factors behind the observed decline in cognitive functions. Menopausal symptoms like vasomotor symptoms, insomnia, and fatigue are also likely to affect the cognitive health of menopausal women (Conde et al., 2021).

Further, the association between cognitive impairment and the higher number of live births may be explained by both biological mechanisms and social/behavioral factors. Pregnancy and childbirth trigger a wide range of alterations in endocrine activities and metabolic processes, including changes in blood lipoprotein level, increased obesity, and central adiposity (Lahmann et al., 2000; Lain and Catalano, 2007; Soma-Pillay et al., 2016). These factors, independently and/or *via* other metabolic and cardiovascular diseases, may be associated with increased odds for CI. Interestingly, some recent studies have highlighted that the higher number of children affects the cognitive health of both men and women, indicating gender indifference (Ning et al., 2020; Song et al., 2020; Gong et al., 2022). Therefore, social/behavioral explanations appear to be more plausible. Having numerous children and raising them in a society with fewer social supports may result in significant psychological stress throughout the reproductive years and beyond, which may have an adverse effect on brain health (Wilson et al., 2006). A higher number of children is also associated with lower economic status and poor education, which, in turn, may affect brain health (Andel et al., 2006).

There are some important limitations of the study that must be mentioned. First, MMSE has been used for CI screening; while it is a reliable screening tool, it is not the gold standard test for diagnosing CI. Second, this is a single-site study; a multi-site study would have been more suitable because environmental factors can modify the relationship between the lipid variables and CI. Another limitation is the lack of hormonal data among women, which would have further substantiated the study’s findings.

## Conclusion and future directions

The present study hints toward a gender-specific relationship between blood lipid parameters (HDL-C in particular) and CI. While none of the blood lipids were found to be associated with CI among males, low HDL-C was protective against CI among females (suggestive significance). Future studies investigating the mechanisms underlying the interactions between plasma lipids and cognition will stimulate new approaches to the treatment and prevention of cognitive disorders. Furthermore, menopause and the higher number of live births were found to be risk factors for CI among females. Pregnancy, childbirth as well as the menopausal transition are crucial reproductive milestones and are associated with significant hormonal changes and stress that may, in turn, be linked to cognitive deterioration.

## Data availability statement

The raw data supporting the conclusions of this article will be made available by the authors, without undue reservation.

## Ethics statement

The studies involving human participants were reviewed and approved by Departmental Ethics Committee, Department of Anthropology, University of Delhi. The patients/participants provided their written informed consent to participate in this study.

## Author contributions

KS and ND designed and executed the research project and conceptualized the manuscript. KK designed and executed the statistical analyses and wrote the first draft of the manuscript. VC and IL reviewed and critiqued the statistical analysis and manuscript preparation. KS, ND, and IL thoroughly reviewed and critiqued the final version of the manuscript. All authors contributed to the article and approved the submitted version.

## Funding

This study was supported by the Research and Development Grant, University of Delhi, New Delhi and Department of Biotechnology, Ministry of Science and Technology, Government of India under grant BT/PRI14378/MED/30/535/2010.

## Conflict of interest

The authors declare that the research was conducted in the absence of any commercial or financial relationships that could be construed as a potential conflict of interest.

## Publisher’s note

All claims expressed in this article are solely those of the authors and do not necessarily represent those of their affiliated organizations, or those of the publisher, the editors and the reviewers. Any product that may be evaluated in this article, or claim that may be made by its manufacturer, is not guaranteed or endorsed by the publisher.
